# Supplementary material for: Reliability of citations of medRxiv preprints in articles published on COVID-19 in the world leading medical journals
Source: PLoS One. 2022 Aug 10;17(8):e0264661. doi: 10.1371/journal.pone.0264661 (PMC9365132; doi:10.1371/journal.pone.0264661)
Supplement: Attachment — Submitted filename: ANswer to reviewers.doc [file pone.0264661.s002.doc]

Answer to reviewers’ comments on the initial manuscript

The two reviewers of this manuscript concur that the paper requires major revision to be reconsidered for publication in PLOS One. I concur with their conclusions and their main points, which are that the paper is scientifically complete, not anchored in relevant literature, has several methodological flaws, and lacks clarity in certain areas. The title is also misleading. The article is more concerned with whether authors of COVID papers follow up on whether cited preprints are later published, than with the fate of the preprints per se.

*We completed the analysis of the results, took into consideration the useful comments of the reviewers and we presented the results more thoroughly.*

*We reviewed all recent articles on preprints, including the ones mentioned by reviewer 2 and we improved the discussion to anchor the manuscript in relevant literature.*

*We agree with the comment on the title and we changed it*

In addition to requesting the improvements identified by the two reviewers, I also request that the authors explicate the concept of ‘preprint’ more fully. The term is a misnomer, dating from an era of paper journals and mimeographed copies of accepted drafts that were disseminated by post to an ‘invisible college.’ The framing of the paper indicates that they are scoping preprint to mean anything in MedRxiv, but they use the term more generically later. As one of the reviewers notes, preprints generally are versions of manuscripts posted at the time of submission to a journal. The manuscript may or may not be published, by that journal or another journal, at some point in time, with numerous changes in form and content along the way. Then what is this malleable entity, and why does it matter ?

*We included this comment in our manuscript and explained the definition of a preprint and we discussed why this term is a misnomer, and why it should not be used.*

*We stated that :*

*“However, preprints posted on preprint platforms are not subject to peer-review, and therefore critical appraisal, until they are submitted to peer-reviewed journals. Only a small proportion will be converted to scientific publications and the share of converted preprints shows a declining trend over time [4–7]. For those finally passing the peer-review process, peer-reviewers’ comments and critiques lead authors to revise their manuscripts, substantially at times [4,6,8]. In that case, the preprint is not the latest version of the work anymore and identifying if it has been published, and referring to the published version instead of the preprint is a major issue as it provides readers with the latest version of a now certified work.”*


Reviewers' comments:

Reviewer #1: This submission analyses the citations of medRxiv preprints in four flagship medical journals. It contributes to the understanding of a recently evolving practice in health sciences: the adoption of preprints as citable items. It stresses some limitations of citing non-peer reviewed literature (some results are updated in subsequent published articles). It shows that the published articles could have been cited instead of preprints for one-third of the citations to preprints that eventually got published. While the title looks descriptive ‘Fate of preprints...’ the authors provide advice for authors, such as search for a potential peer-reviewed article before citing a paper as the former might better reflect the latest evidence available.


I understand from the authors' statement (l20) that they had a ‘letter’ format in mind yet the article type is ‘Research Article’ in the PDF for review. This should be clarified.

*Although we had initially a letter in mind, we chose to develop it as a full article.*


I listed my comments chronologically below -- except from the first item on data availability which seems most critical to me.


* l186: “Availability of data and method: The datasets used and/or analysed during the current study are available from the corresponding author on reasonable request.”

This does not comply with PLOS's policy.
The PDF I received for review reads as follows (page 5): “Important: Stating ‘data available on request from the author’ is not sufficient. If your data are only available upon request, select ‘No’ for the first question and explain your exceptional situation in the text box.”

See also <https://journals.plos.org/plosone/s/data-availability>

I advise the authors to upload their (valuable hand-collected) dataset to [https://zenodo.org](https://zenodo.org/) and provide the DOI any revised version of the manuscript

*We uploaded dataset in zenodo :*

[***https://doi.org/10.5281/zenodo.5985960***](https://doi.org/10.5281/zenodo.5985960)

* l1: The title should be revised to specify ‘MedRxiv’ as the study focused on this preprint and several others hosted COVID19 research (Fraser et al., 2021; Kirkham et al., 2020) <-- Two overlooked relevant references for this paper.

*The title was changed: Reliability of citations of medRxiv preprints in articles published on COVID-19 in the world leading medical journals.*

* l25: ‘The COVID-19 pandemic has led to an exponential growth of preprints’ -> unsubstantiated. Refer to a tide/wave or any metaphor (Brainard, 2020) but not to a mathematical model please. The use of the exponential model for preprints is inaccurate, production is closer to a linear model (see bottom figure of <https://github.com/nicholasmfraser/covid19_preprints>).

*This sentence was changed:*

*“The scientific community has reacted rapidly to the medical challenges generated by the coronavirus disease 2019 (COVID‐19) pandemic, with a steady increase of literature in peer-reviewed journals but also in preprint repositories [1–3]. Yet, a steep increase in the number of posted preprints was observed between January and December 2020, from 797 to 14 290, out of which 8858 (62.0%) were COVID‐19‐related [4].”*

* l26: ‘and will never been published for most of them’ -> hearsay, please cite your sources.

*This sentence was changed and many studies were cited*

*“Only a small proportion will be converted to scientific publications and the share of converted preprints shows a declining trend over time [4–7]. For those finally passing the peer-review process, peer-reviewers’ comments and critiques lead authors to revise their manuscripts, substantially at times [4,6,8]. In that case, the preprint is not the latest version of the work anymore and identifying if it has been published, and referring to the published version instead of the preprint is a major issue as it provides readers with the latest version of a now certified work.”*


* l27: ‘most prestigious journals’ -> subjective concept to be defined

*We changed the sentence and explained why we chose these journals*

*“Articles on COVID have been published in many different journals but we chose to investigate the BMJ, The Lancet, the JAMA and the NEJM because they are the medical journals that have published much of the research on Covid-19 [12,13].”*

* l31: ‘the reference lists ... were manually screened’ -> some authors do not properly cite preprints but put the hyperlink in a footnote. Does your method account for this unconventional citing behaviour?

*We clarified the sentence. We searched for in-text citations as foot-notes and in the reference list of all articles*

* l68: ‘preprints are now used’ -> please clarify as one can use preprints in multiple ways: I can post a preprint, cite a preprint, text-mine a preprint...

*This mention was removed*

* l69: ‘to assess the use of MedRxiv preprint’ -> use as cited references

*We changed the description of objectives:*

*The first objective of our study was to assess the reliability of preprint citations in articles on COVID-19 published by the world leading medical journals. The second objective was to assess the rate of publication of preprints cited in articles appearing in theses journals and the last objective was to compare, if relevant, the content of the preprints to their published version.*

* l93: only one assessor so no inter-rater agreement reported (Artstein & Poesio, 2008). This is unusual and should at least be stressed as a limitation of the study design.

*We agree with this comment. We re-analyse the data, and updated the result section*

*Two authors (JFG and LR) compared the data between preprints and their published version. Disagreement was resolved by consensus*

* l93: ‘by the same evaluator’ -> was the evaluator an expert of the field? Please clarify.

*JFG and LR have published several studies on bibliometrics*. *JFG is expert in the COVID Group of the French High Council of Public Health.*

* l100: ‘(p=0.04)’ which test? This does not comply with how statistics should be reported, see <https://journals.plos.org/plosone/s/submission-guidelines> and <https://www.equator-network.org/wp-content/uploads/2013/03/SAMPL-Guidelines-3-13-13.pdf>

*This comparison had no added value and we removed it*

* l105: ‘before September’ -> +2020

*We clarified this point*

*All research articles published on COVID-19 between the 1st of January and the 1st of December 2020 in the British Medical Journal (BMJ), The Lancet, the Journal of the American Medical Association (JAMA) and the New England Journal of Medicine (NEJM) were identified through the journal websites. We then manually screened these articles to identify all articles citing at least one preprint from MedRxiv, in the text as a footnote but also in the reference list since all these journals allow citation of preprints in the reference list of articles they published.*

* l109: Only 45.4% of the published preprint were reported as such in MedRxiv. This confirms the observation of (Cabanac et al., 2021) and should be clarified.

*This point was discussed more thoroughly*

* l136: Authors found that preprints and published versions differ. It may be worth saying that this is expected for preprints that serve as drafts.

*This point was discussed more thoroughly, with the citation of many different studies*

*“For the preprints which were finally published, the differences between the preprints and their published version were frequent and close to those reported in another study on 139 preprints, with changes in the study results or the abstract conclusions between the first preprint version to journal article for 38% and 29% of preprints, respectively [6]. However, when studying 67 studies on Covid-19 prevention or treatment, Bero et al. found only 23 articles (34%) that had no discrepancies in results reporting between preprints and journal publications [8].*

*The changes in the conclusions were less frequent than the changes in studies’ result, mostly because conclusions were usually broad, such as “among patients hospitalized with Covid-19, those who received hydroxychloroquine did not have a lower incidence of death at 28 days than those who received usual care” [22].*

*These changes can be the consequence of publishing preliminary results in a preprint and final results in a peer-reviewed journal, of the fact that some preprints receive comments that allow authors to improve their manuscript prior to submission to a journal, or of the improvement of the quality of the report following the peer reviewed process.*

*Preprints on COVID-19 posted in preprint servers are shorter and contain less references than non-COVID-19 preprints, which suggests that authors tend to publish preliminary results [1].*

*Concerning gradual quality improving, although preprint servers offer authors the opportunity to post updated versions of a preprint, enabling them to incorporate feedback, correct mistakes, or add additional data and analysis, the majority of preprints on COVID-19 in preprint servers exist in fact as only a single version [1]. A study on bioRxiv preprints showed that less than 10% of preprints received at least one comment, and one third were posted by the preprint’s authors themselves [23].”*


* l167: The authors could checked if some cited preprints had been commented on PubPeer and/or withdrawn from the servers (this happens <https://retractionwatch.com/2021/05/17/preprints-are-works-in-progress-the-tale-of-a-disappearing-covid-19-paper/>).

*We discussed this point, referring to previous studies on that topic :*

*“None of the preprints cited were withdrawn from medRxiv server.”*

*“Concerning gradual quality improving, although preprint servers offer authors the opportunity to post updated versions of a preprint, enabling them to incorporate feedback, correct mistakes, or add additional data and analysis, the majority of preprints on COVID-19 in preprint servers exist in fact as only a single version [1]. A study on bioRxiv preprints showed that less than 10% of preprints received at least one comment, and one third were posted by the preprint’s authors themselves [23].”*


* Typos

l26: ‘will never been published’ -> be

l63: ‘preprints are’ -> were

*The corrections were made*

* References

Artstein, R., and Poesio, M. (2008), "Inter-Coder Agreement for Computational Linguistics", Comput. Ling., 34(4):555-596. <https://doi.org/10.1162/coli.07-034-R2>

Brainard, J. (2020). Scientists are drowning in COVID-19 papers. Can new tools keep them afloat? Science. <https://doi.org/10.1126/science.abc7839>

Cabanac, G., Oikonomidi, T., & Boutron, I. (2021). Day-to-day discovery of preprint–publication links. Scientometrics, 126(6), 5285–5304. <https://doi.org/10.1007/s11192-021-03900-7>

Fraser, N., Brierley, L., Dey, G., Polka, J. K., Pálfy, M., Nanni, F., & Coates, J. A. (2021). The evolving role of preprints in the dissemination of COVID-19 research and their impact on the science communication landscape. PLOS Biology, 19(4), e3000959. <https://doi.org/10.1371/journal.pbio.3000959>

Kirkham, J. J., Penfold, N. C., Murphy, F., Boutron, I., Ioannidis, J. P., Polka, J., & Moher, D. (2020). Systematic examination of preprint platforms for use in the medical and biomedical sciences setting. BMJ Open, 10(12), e041849. <https://doi.org/10.1136/bmjopen-2020-041849>

Reviewer #2: The paper is of interest to the research community and presents some interesting findings that I would like to see published. However, in its present format, it does not have the attributes of a research article. The article lacks a literature review, a list of hypotheses, a discussion that engages with past work on this topic and discusses how this article is advancing knowledge on the subject (right now the discussion reads like a "limitations section"), and a proper conclusion. At the moment, the paper reads overall like a short public report.

I see two possible solutions here. If the authors want to publish it as a research article, they have to conduct much more research in order to sustain and properly discuss their findings. In particular, they should engage with key literature on the role of preprints from the fields of information science, science of science, scholarly communications and science and technology studies (at least). Happy to provide further guidance on this, if the authors decide to take this direction. Another possibility – probably easier and faster – is that the authors re-write this piece as a commentary or a research note of some sort.

*We thank the reviewer for his valuable mention of these papers. We included them and reviewed all those published on preprint since then which allowed to o improve the discussion.*

Another major issue with the paper is the language. The paper needs to be heavily edited for clarity. Some examples:

-Line 26: The authors say "(preprints) are not subject to peer-review" but they actually mean that when preprints are published on preprint servers they are not peer-reviewed YET, but then they are indeed subject to peer-review (even if some of them won't be published)

*We fully agree and we discussed this point. However, a preprint is not, per se, subject to peer review. When it passes the peer-review process, it is not a preprint anymore, and should not be referred as such.*

*We added in the background section “However, preprints posted on preprint platforms are not subject to peer-review, and therefore critical appraisal, until they are submitted to peer-reviewed journals »*

-Line 27: Preprints are "used and cited". What's the difference between used and cited? I think the authors just mean cited here.

*We removed the mention to the use of preprints*

-Line 28/29/30: This sentence has to be re-written. I can intuitively understand the subject-object-verb relations but the writing is not clear at the moment

*We changed the description of methods:*

*Articles published on COVID in 2020 in the BMJ, The Lancet, the JAMA and the NEJM were manually screened to identify all articles citing at least one preprint from medRxiv. We searched PubMed, Google and Google Scholar to assess if the preprint had been published in a peer-reviewed journal, and when. Published articles were screened to assess if the title, data or conclusions were identical to the preprint version*

-Line 40: "Among them" Among which articles?

*We mentioned : “Among the 182 preprints »*

Line 41: "...39 had in fact already been published in a peer reviewed journal at the time of online publication of the citing article" What does this mean and why is it interesting? Is this saying that author X cited in article X a preprint Y when Y was a preprint, and by the time article X was published, preprint Y became a peer-reviewed publication? Or does it mean something else? If so, why is this interesting? (the authors mention this finding a couple of times in the paper)

*This is exactly the point and we discussed that. This is interesting because there are differences between the preprint cited and its published version.*

*“Referring to the published version instead of the preprint is a major issue as it provides readers with the latest version of a now certified work »*

-Line 68: "preprints are used" do you mean cited?

*We changed it*

-Line 74: "until 1st of December 2020" - when did you start the analysis?

*We gave more precision in the method section*

*« All research articles published on COVID-19 between the 1^st^ of January and the 1^st^ of December 2020 …»*

-Line 117/118 "at the time of the study" which study?

*We made this point clearer, which was not the case in this sentence*

*We replaced*

*“Among the 74 articles that were not published in a peer-reviewed journal at the time of the study, 68 (91.9%) had been posted in MedRxiv more than 28 days before the online publication of the citing article, 49 (66.2%) more than 46 days and 41 (55.4%) more than 72 days.”*

*By*

*“However, among those 124 “preprints”, 51 (41·1%) had already been published when the citing article was published online, at least 2 months before for 33 of them, and were, in fact, not preprints anymore at that time.”*
