# Supplementary material for: Reliability of citations of medRxiv preprints in articles published on COVID-19 in the world leading medical journals
Source: PLoS One. 2022 Aug 10;17(8):e0264661. doi: 10.1371/journal.pone.0264661 (PMC9365132; doi:10.1371/journal.pone.0264661)
Supplement: Attachment — Submitted filename: Rebutal letter July 2022.docx [file pone.0264661.s003.docx]

PONE-D-22-04047

Rebutal letter July the 3^rd^, 2022

Reviewers' comments:

Reviewer #1: I agree with the other reviewers that this could go as a commentary rather than a full research article. Since the analysis is not complex and does not involve sufficient examples, for example across multiple platforms, the conclusions reached cannot be generalized. At the same time, the authors have drawn attention to an interesting problem arising from the citation of non-peer reviewed work in published papers. The authors have also mentioned some cautionary measures.
I believe this paper should be published.

Some minor changes in language ->
71 May like to use “important issue” in place of “major…”….
In the following, X denotes crossed out.
73 Yet …Remove
75 Nevertheless…Remove
85 11 months? …or should it be 31 December?
113 Overall , 182 UNIQUE preprints….?
234 a Remove
248 shortcoming -> shortcomings

With thank reviewer #1 for his general comments on the manuscript and for the changes in language he suggested.

All the changes have been made in the revised manuscript.
